# Heparan Sulfate Mimetics: A New Way to Optimize Therapeutic Effects of Hydrogel-Embedded Mesenchymal Stromal Cells in Colonic Radiation-Induced Damage

**DOI:** 10.1038/s41598-018-36631-6

**Published:** 2019-01-17

**Authors:** Lara Moussa, Christelle Demarquay, Gildas Réthoré, Mohamed Amine Benadjaoud, Fernando Siñeriz, Girish Pattapa, Jérôme Guicheux, Pierre Weiss, Denis Barritault, Noëlle Mathieu

**Affiliations:** 1 IRSN, Institut de Radioprotection et de Sûreté Nucléaire, PSE-SANTE, SERAMED, LRMed, 31 avenue de la division Leclerc, 92262 Fontenay-aux-Roses, France; 2 INSERM, Institut National de la Santé et de la Recherche Médicale, U1229, Regenerative Medicine and Skeleton (RMeS), Faculté de Chirurgie Dentaire, 1 Place Alexis Ricordeau, 44042 Nantes, France; 3 Université de Nantes, Regenerative Medicine and Squeleton (RMeS), Faculté de Chirurgie Dentaire, 1 Place Alexis Ricordeau, 44042 Nantes, France; 4 Centre Hospitalier Universitaire de Nantes, Pôle Hospitalo-Universitaire 4 (OTONN), 1 Place Alexis Ricordeau, 44042 Nantes, France; 5 Société OTR3 (Organes, Tissus, Régénération, Réparation, Remplacement), 4 Rue Française, 75001, Paris France; 6 Université Paris-Est Créteil, Laboratoire de recherche sur la Croissance Cellulaire, Réparation, et Régénération Tissulaire, Faculté des Sciences, Université Paris-Est Créteil, 61 Ave du Gal de Gaulle, 94000 Créteil, France

## Abstract

Clinical expression of gastrointestinal radiation toxicity on non-cancerous tissue could be very life threatening and clinicians must deal increasingly with the management of late side effects of radiotherapy. Cell therapy, in particular mesenchymal stromal cell (MSC) therapy, has shown promising results in numerous preclinical animal studies and thus has emerged as a new hope for patient refractory to current treatments. However, many stem cell clinical trials do not confer any beneficial effect suggesting a real need to accelerate research towards the successful clinical application of stem cell therapy. In this study, we propose a new concept to improve the procedure of MSC-based treatment for greater efficacy and clinical translatability. We demonstrated that heparan sulfate mimetic (HS-m) injections that restore the extracellular matrix network and enhance the biological activity of growth factors, associated with local injection of MSC protected in a hydrogel, that increase cell engraftment and cell survival, improve the therapeutic benefit of MSC treatment in two animal models relevant of the human pathology. For the first time, a decrease of the injury score in the ulcerated area was observed with this combined treatment. We also demonstrated that the combined treatment favored the epithelial regenerative process. In this study, we identified a new way, clinically applicable, to optimize stem-cell therapy and could be proposed to patients suffering from severe colonic defect after radiotherapy.

## Introduction

Radiotherapy is an indisputable tool for the management of malignant pelvic diseases. The aim of radiotherapy is to deliver sufficient doses of ionizing irradiation to destroy the tumor and prevent the recurrence of the malignancy. Even though much progress has been made in treatment delivery techniques, external radiotherapy leads to the exposure of healthy tissues surrounding the tumor. Because it rapidly renews itself, intestinal tissue is particularly radiosensitive and its presence in the irradiation field is a limiting factor for abdominal and pelvic radiotherapy protocols.

Intestinal radiation toxicity is a complex process involving inflammation, epithelial stem-cell death, and activation of endothelial cells and immune system associated with persistent oxidative stress. Altogether, these processes lead to a loss of the intestinal barrier resulting in the entry of immunogenic substances which worsen the inflammatory state of the tissue and prevent the tissue to renew itself^1^. The mechanism of radiation-induced injury is a continuous process starting immediately after irradiation which can last and exacerbate over the years. This progressive process is characterized by a self-maintained scaring process with excessive accumulation of extracellular matrix (ECM) component, mainly collagen, inducing a defective healing process often causing loss of tissue function. In the worst cases, transmural fibrosis may progress to colonic obstruction^2^.

In recent years, treatment of malignant tumors has achieved satisfying efficacy. The number of cancer survivors is increasing each year and, therefore, the associated normal tissue radio-induced toxicity. It has been evaluated that almost twice as many patients develop substantial gastrointestinal problems after pelvic radiotherapy as are diagnosed annually with Crohn’s disease. 5 to 10% of patients treated with radiotherapy for abdominal cancers develop severe complications 5 to 10 years after the end of the treatment respectively. These chronic side effects adversely affect their quality of life and, in some cases, can be very incapacitating. Some pharmacological molecules have been tested, such as pravastatin and pentoclo, however, none has demonstrated a curative effect in repairing radiation-induced lesions and their clinical effect need to be further investigated^3,4^. The only option for the patient is the surgical resection of the fibrosed part of the colon. Healing of colonic anastomoses after surgery is difficult under normal conditions and is extremely compromised following irradiation. The major surgical problem is the dehiscence of tissues causing a leakage of the anastomosis. This wound healing failure could be due to excessive inflammation and impaired ECM induced after irradiation.

Convincing experimental results from different animal models and on clinical cases have shown that mesenchymal stromal cell (MSC) treatment promotes therapeutic benefit in various diseases. MSC injections have been given on compassionate grounds to treat radiation-induced proctitis in patients who were over-irradiated after a radiotherapy accident. Three patients suffering from rectal bleeding and pain were systemically given within-family allogeneic MSCs. Although it was difficult to assess the treatment because of its compassionate nature, positive results were achieved in terms of pain reduction in two patients and a reduction in the number of episodes of diarrhoea and cessation of bloody discharge in one patient^5,6^.

Preclinical studies have also demonstrated that intravenous (IV) injection of MSCs reduces severe colorectal lesions caused by ionizing irradiation by promoting epithelial regeneration, improving the intestinal structure and modifying the inflammatory process^7–11^. Despite the large number of systemically injected cells (15.10^6^ cells/kg), few cells could be detected in the colonic mucosa^7^. At the time, various studies suggested that the beneficial effects of MSCs were related to their secretion of bioactive molecules^12,13^. Indeed, in this sense, we recently demonstrated that a local injection of MSCs within a hydrogel (Silanized HydroxyPropylMethyl Cellulose Si-HPMC) through colonoscopy allows a decrease of the number of injected cells, increases the rate of MSC engraftment in the area requiring repair and improves the function of epithelial barrier in the colon^14^. However, the radio-induced ulcer is persistently associated with numerous inflammatory cells that could limit the regenerative process. The high proteolytic activity of these cells could participate in the degradation of ECM and growth factors required for wound healing.

A growing number of observations have demonstrated that heparan sulfates (HS) are a key component of the ECM, via their ability to act both as a scaffold element bridging matrix proteins through their heparin binding sites and as a reservoir of growth factors, which protects and regulates the bioavailability of communication factors. This ECM network plays important roles in homeostasis and remodeling. Following injury, the destruction of HS contributes to spatial disorganization of the ECM and contributes to the impoverishment of bioactive molecules in the injured tissue. Importantly, delivery of growth factors is not sufficient for healing if they cannot be retained and presented to their respective receptor. Matrix therapy is based on the concept of restoring the cellular microenvironment with the aim of protecting and promoting communication between cells. Many studies have demonstrated the benefits of heparan sulfate mimetics (HS-m) named RGTA^®^ in improving tissue healing in numerous pathological contexts and tissues such as the skin, cornea, bone, joint, tendon and muscle^15^. Although structurally and functionally analogous to naturally derived HS, one of the most crucial properties of RGTA^®^ is its resistance to glycanase degradation. This allows RGTA^®^ to retain their structure and activity even in the microenvironment of a chronic wound.

In this study, we aimed to evaluate the use of an HS-m to improve hydrogel-assisted cell therapy using MSCs from adipose tissue in an animal model developing severe colonic radio-induced damages after local irradiated through a window centered on the colon^7^. We used a dedicated HS-m molecule namely RGTA^®^, injected intravenously, to facilitate reconstruction of the ECM scaffold, a necessary first step in reestablishing a microenvironment able to host MSC-secreted factors. The therapeutic potential of this combination was tested on two different models, representative of clinical cases found in patients developing severe side effects after radiotherapy^7^. First, the rats were treated 3 weeks after irradiation and the therapeutic benefit was evaluated on the structure and the function of barrier at four weeks. Second, 3 weeks after irradiation, colonic anastomosis was performed. The treatment was applied for 5 weeks, the survival of animals and the quality of the anastomosis structure by collagen-ECM composition were evaluated.

## Results

### Effects of HS-mimetics (HS-m) on colonic epithelial structure and epithelial barrier function

**Figure 1:**
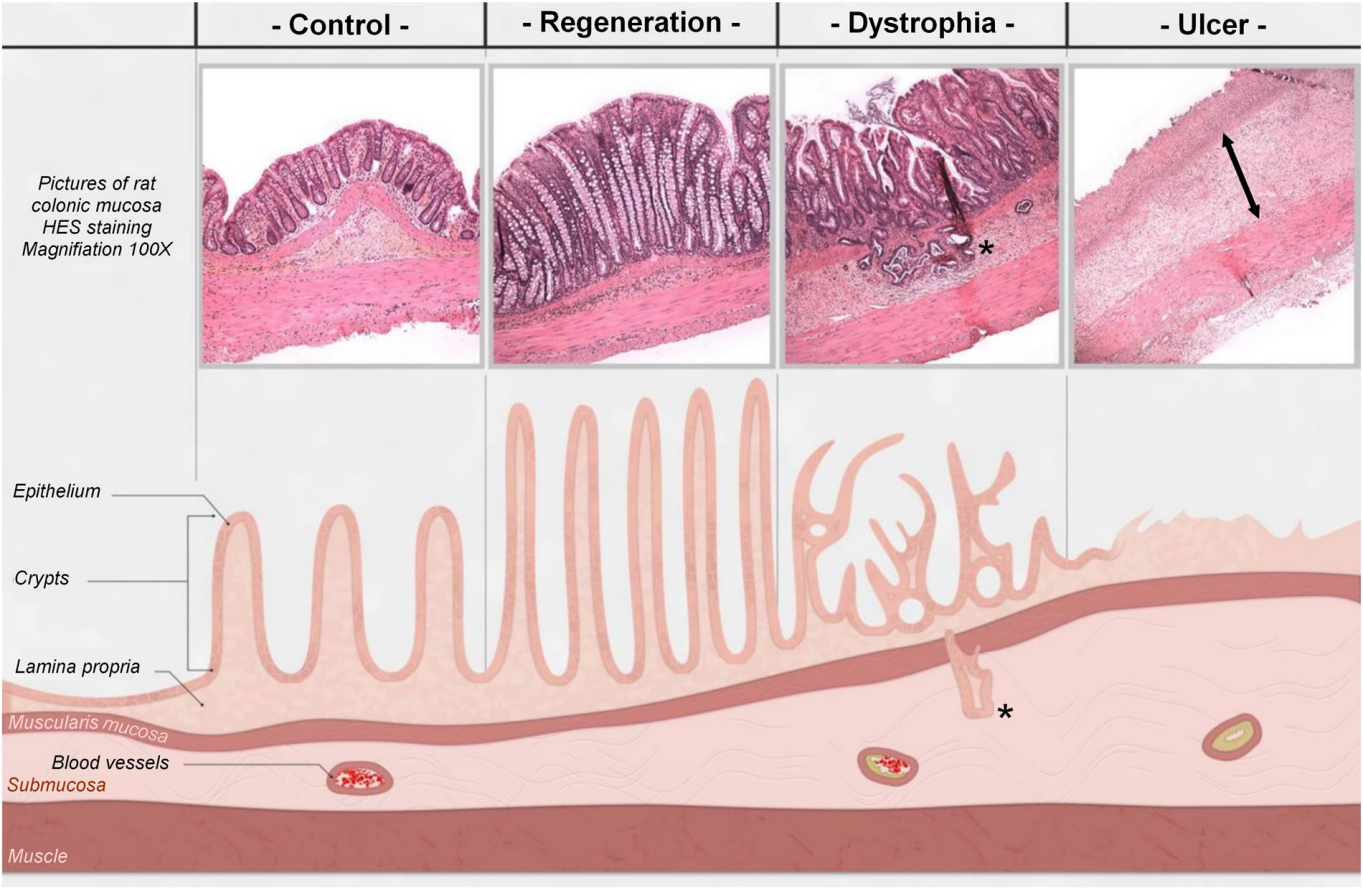
Representative histological pictures and schematic illustration of the colonic lesion induced after irradiation (4 weeks). In control (non-irradiated) animal, crypts are organized and aligned on the muscularis mucosa. The irradiated area was separated into three zones: (1) The regenerative zone with elongated and organized crypts. (2) The dystrophic zone, characterized by the presence of disorganized crypts with a small edema. Colitis cystica profunda(*) could appear in the most inflamed area. (3) The ulcerated zone where crypts were totally absent and replaced by a dense inflammatory infiltrate with an important edema (<-> arrows) and extracellular matrix remodeling.

**Figure 2:**
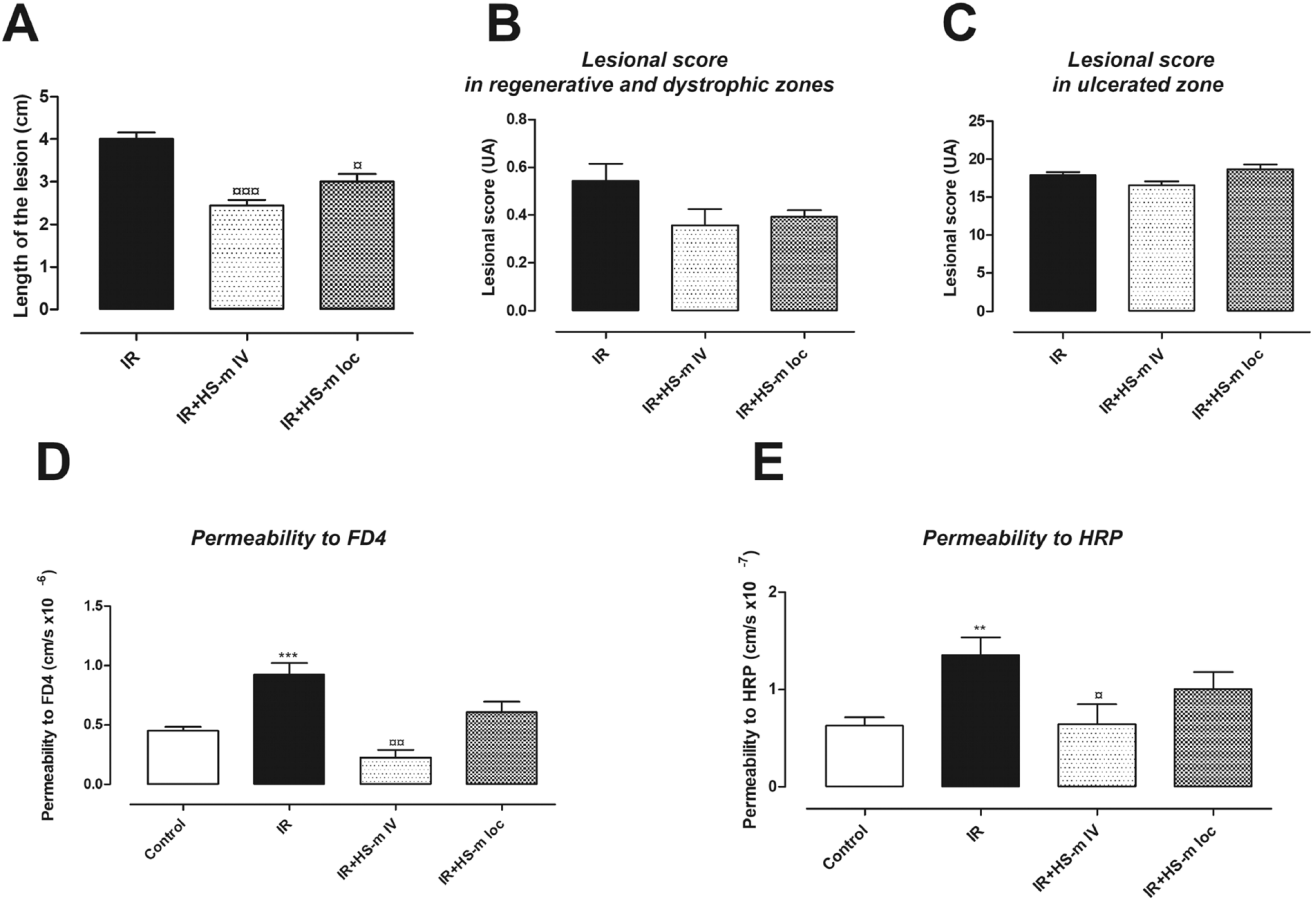
Effects of HS-mimetic on radiation-induced colonic damage 4 weeks after irradiation. HS-m was injected locally or intravenously 3 weeks after irradiation to the rats as described in protocol 1 of the Supplementary Fig. 2. (**A**) Length of the lesion (macroscopic score) (**B**,**C**) Lesional score in regenerative, dystrophic and ulcerated areas (**D**,**E**) Measurement of colonic permeability to FD4 and HRP respectively using Ussing Chambers. N = 10 animals per group. IR = irradiated rats (4 weeks), IR + HS-m IV = irradiated rats IV injected with HS-m, IR + HS-m loc = irradiated rats locally injected with HS-m, control = non-irradiated rats. ^**^p < 0.01 vs. control, ^***^p < 0.001 vs. control, ^¤^p < 0.05 vs. IR rats, ^¤¤^p < 0.01 vs. IR rats, ^¤¤¤^p < 0.001 vs. IR rats.

The epithelial damage induced by local irradiation of the rat colon was evaluated on structural and functional parameters over time (Supplementary Fig. 1). Four weeks after irradiation, both of these parameters were modified. At this time, histologic analysis revealed colonic inflammation associated with crypt atypia, ulceration and bowel wall thickening as observed in patients developing late side effects of radiotherapy. Representative histological pictures are illustrated in Fig. 1. Therapeutic dose-effect of HS-m has been previously established^16^, however it has been necessary to test the best way of injection in our model. Thus, intravenous injection and local injection in colonic mucosa of HS-m have been tested (Scheme of the procedure in Supplementary Fig. 2). First, macroscopic damage was evaluated directly on the colonic tissue and visualized as white and thick mucosa. We observed that administration of HS-m treatment significantly decreased the length of damaged tissue irrespective of the way of injection, compared with irradiated animals (Fig. 2A). Then, we quantified the severity of the lesion on histologic slides 4 weeks after irradiation. The irradiated zone can be separated into three areas: regenerative, dystrophic and ulcerated areas (Fig. 1). Inside these specific zones, injury score taking into account mucosal damage, edema, vascular sclerosis and muscular dystrophy was evaluated. We demonstrated that irradiated rats treated with HS-m exhibit slight decrease of the injury score in the regenerative and dystrophic areas compared with irradiated rats (Fig. 2B). However, no effect was shown after HS-m treatment in the ulcerated area in both injections protocol (Fig. 2C).

The intestinal barrier protects the organism against the invasion of luminal microorganisms and pathogens; however, it should be open to absorb fluids and nutrients. This opposing role is maintained by a complex anatomical and functional structure described as “intestinal permeability”. Here, the influence of HS-m injection on the colonic permeability to FD4 (fluoro isothiocyanate (FITC)-Dextran 4 kDa) and intact HRP (Horseradish peroxidase) was examined in Ussing chambers. Four weeks after irradiation, the colonic permeability to FD4 and to intact HRP was significantly increased compared with control rats (Fig. 2D,E). This study determined that injection of HS-m by intravenous route significantly decreased irradiation-induced hyperpermeability to FD4 and HRP (Fig. 2D,E). However, these parameters were not statistically modified by HS-m injected locally; consequently the IV injection has been chosen for the following experiments.

### Improvement of the therapeutic benefit induced by hydrogel-assisted MSC therapy with HS-m co-treatment

**Figure 3:**
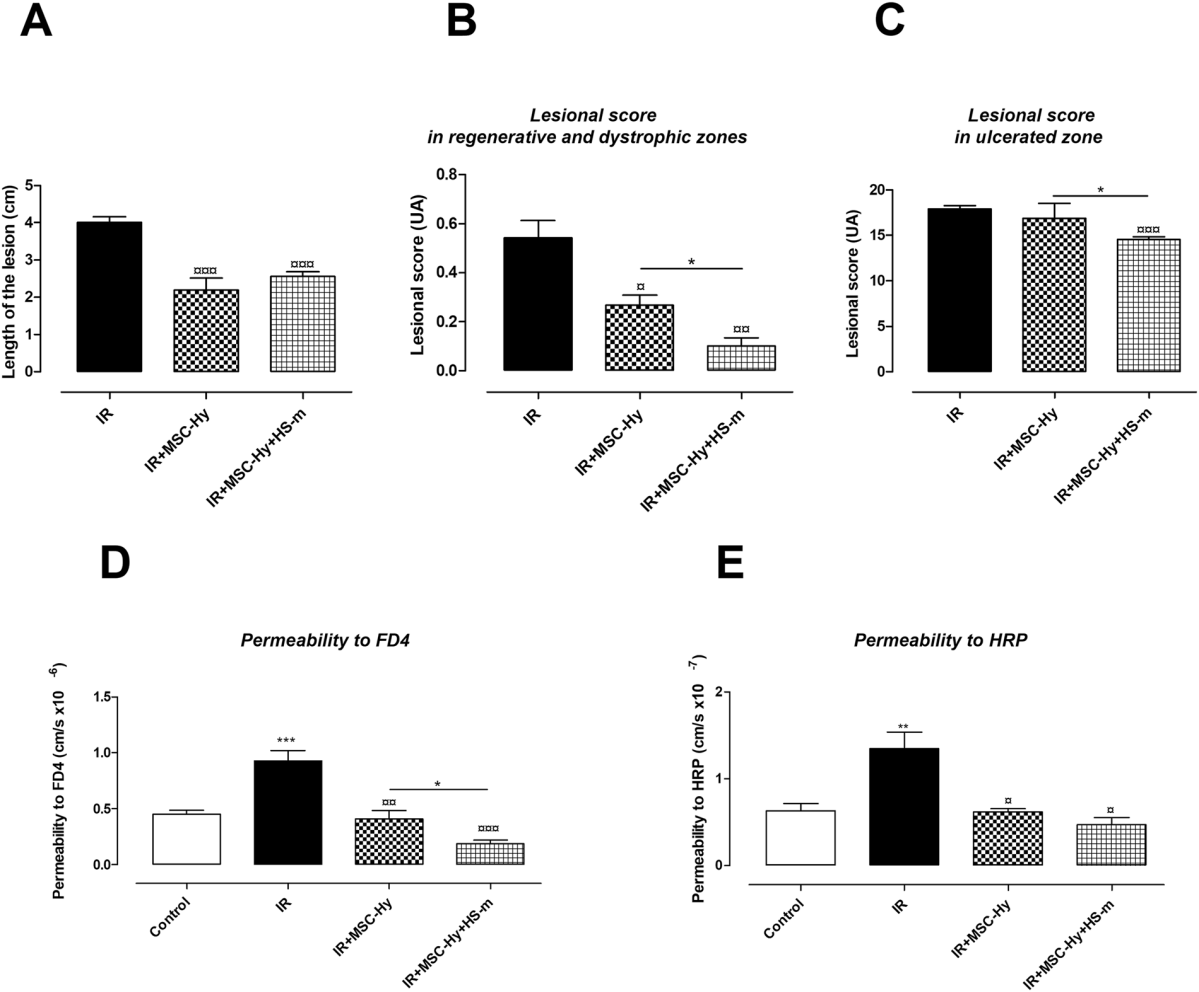
Improvement of the therapeutic benefit induced by hydrogel-assisted MSC therapy with HS-m co-treatment (4 weeks after colonic irradiation). HS-m and MSC-hy were injected into the rats as described in the protocol 1 of Supplementary Fig. 2. (**A**) Length of the lesion (macroscopic score). (**B**,**C**) Lesional score in regenerative, dystrophic and ulcerated areas. (**D**,**E**) Measurement of colonic permeability to FD4 and HRP respectively using Ussing Chambers. N = 10 animals per group. IR = irradiated rats (4 weeks), IR + MSC-Hy = irradiated rats treated with hydrogel-embedded MSCs, IR + MSC-Hy + HS-m = irradiated rats treated with hydrogel-embedded MSCs + HS-m, control = non-irradiated rats. ^**^p < 0.01 vs. control, ^***^p < 0.001 vs. control, ^¤^p < 0.05 vs. IR rats, ^¤¤^p < 0.01 vs. IR rats, ^¤¤¤^p < 0.001 vs. IR rats, ^*^p < 0.05 IR + MSC-Hy vs. IR + MSC-Hy + HS-m.

We previously demonstrated the therapeutic benefit of MSCs embedded in Si-HPMC hydrogel^14,17^. In order to improve the therapeutic benefit of MSC therapy on severe lesion, we combined IV injection of HS-m with hydrogel-assisted cell therapy (Scheme of the procedure in Supplementary Fig. 2). First of all, we verified the phenotype and the capacities of differentiation of injected-MSCs (Supplementary Fig. 3). We also checked that HS-m does not affect the viability and activity of MSCs (Supplementary Fig. 4). Then, *in vivo* analyses were realized and demonstrated that at macroscopic level, hydrogel-assisted MSC therapy (MSC-Hy) with or without HS-m injection decreased colonic damage (Fig. 3A). Moreover, histological analyses revealed a more pronounced reduction of the epithelial injury score in the regenerative and dystrophic areas with HS-m co-treatment (Fig. 3B). Most interestingly, the injury score was significantly decreased in the ulcerated zone in rats treated with MSC-Hy + HS-m compared to rats treated with MSC-Hy (Fig. 3C).

We also assessed whether this combination could improve the colonic hyper-permeability induced after irradiation. We demonstrated that the reduction in the colonic permeability to FD4 was more pronounced when animals were treated with MSC-Hy + HS-m compared to rats treated with MSC-Hy (Fig. 3D). MSC-Hy treatment reduced colonic permeability to intact HRP to control level, and injection of HS-m did not modify this parameter (Fig. 3E).

### Combined treatment increases the proliferation of crypt epithelial cells after irradiation

**Figure 4:**
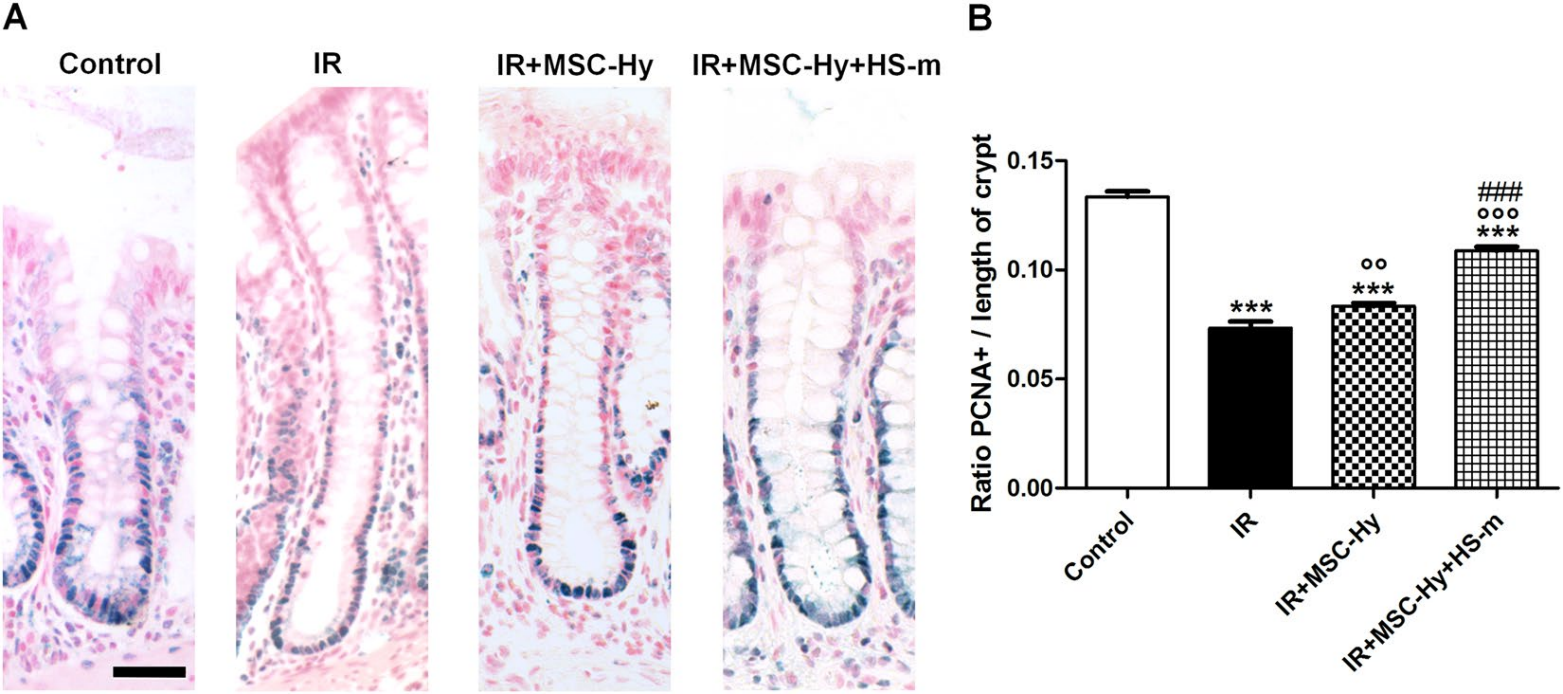
Combined treatment stimulates epithelial proliferation reduced by irradiation (4 weeks). (**A**) Representative pictures of PCNA immunostaining (blue staining) on colonic crypts in the different groups. Scale bars represent 50 µm. (**B**) Quantification of PCNA-positive cells per length of crypt. N = 8 animals per group. Control = non-irradiated rats, IR = irradiated rats (4 weeks), IR + MSC-Hy = irradiated rats treated with hydrogel-embedded MSCs, IR + MSC-Hy + HS-m = irradiated rats treated with hydrogel-embedded MSCs + HS-m IV injected. ^***^p < 0.001 vs. control, ^¤¤^p < 0.01 vs. IR rats, ^¤¤¤^p < 0.001 vs. IR rats, ^###^p < 0.001 vs. IR + MSC-Hy

Epithelial proliferation is an essential component in the colonic regeneration process. To further decipher the role of epithelial proliferation in the regenerative process after the combined treatment, we assessed PCNA (Proliferating Cell Nuclear Antigen) immunostaining. The ratio of PCNA-positive cells per length of the crypt was evaluated (Fig. 4). Four weeks after irradiation, the number of proliferating cells, located in the regenerative area, decreased compared to the control group. In irradiated-MSC-Hy treated rats the number of proliferating cells per crypt length is increased compared with irradiated rats and this number is further improved in combined-treated rats (Fig. 4).

**Figure 5:**
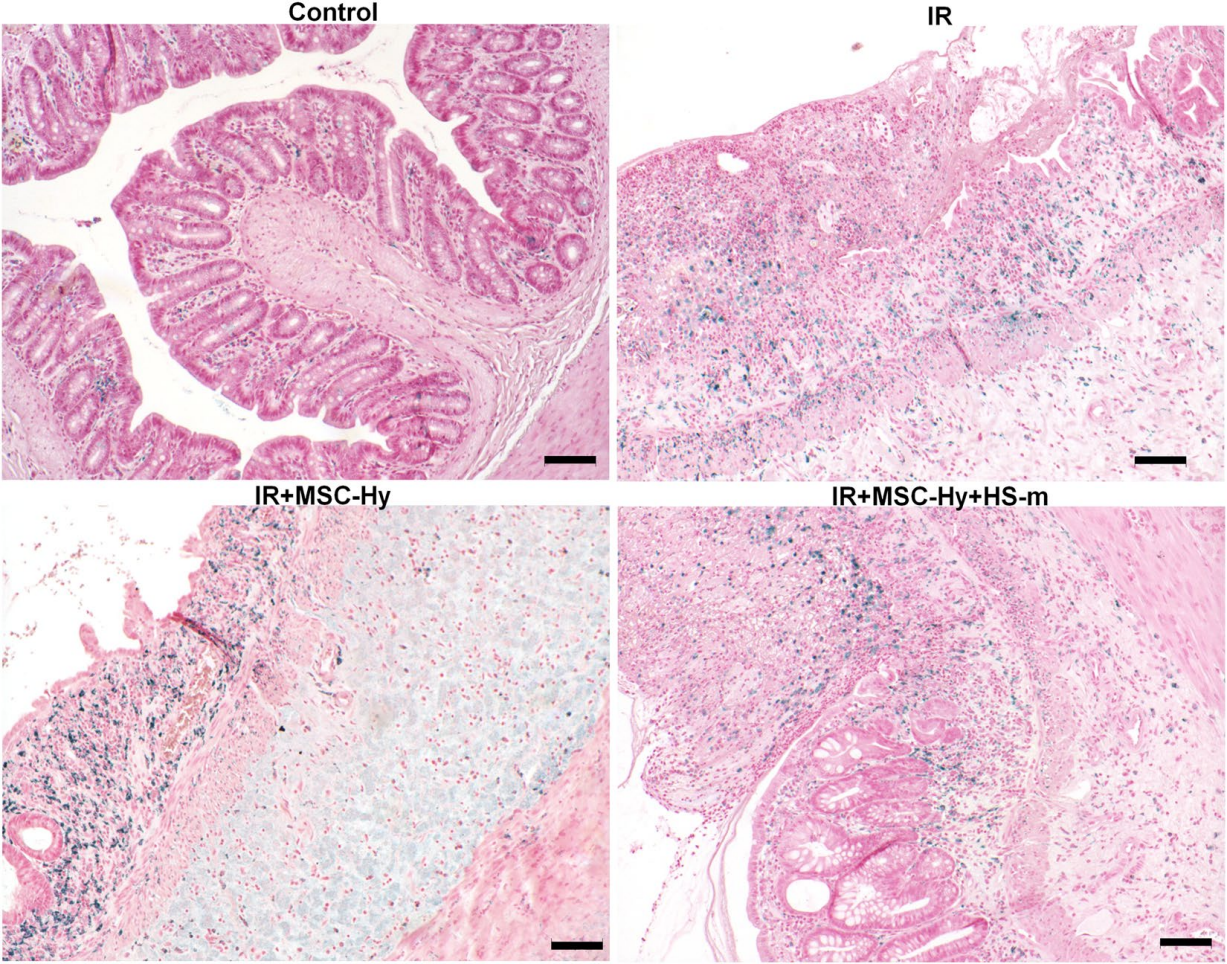
Inflammatory cell infiltrate is not modified after treatments. (**A**) Representative pictures of macrophages immunostaining using CD68 antibody (blue staining). Control = non-irradiated rats, IR = irradiated rats (4 weeks), IR + MSC-Hy = irradiated rats treated with hydrogel-embedded MSCs, IR + MSC-Hy + HS-m = irradiated rats treated with hydrogel-embedded MSCs + HS-m IV injected. Scale bars represent 100 µm.

Radio-induced inflammation takes an important role in the development of the pelvic radiation disease. Indeed, important inflammatory cell infiltrate has been described in patients. We analyzed modification of macrophage infiltrate in the colonic mucosa with the various treatments; however we were not able to detect statistical modification with irradiated animals between the different groups of treated animals (Fig. 5).

### Determination of the colonic anastomosis procedure after irradiation

**Figure 6:**
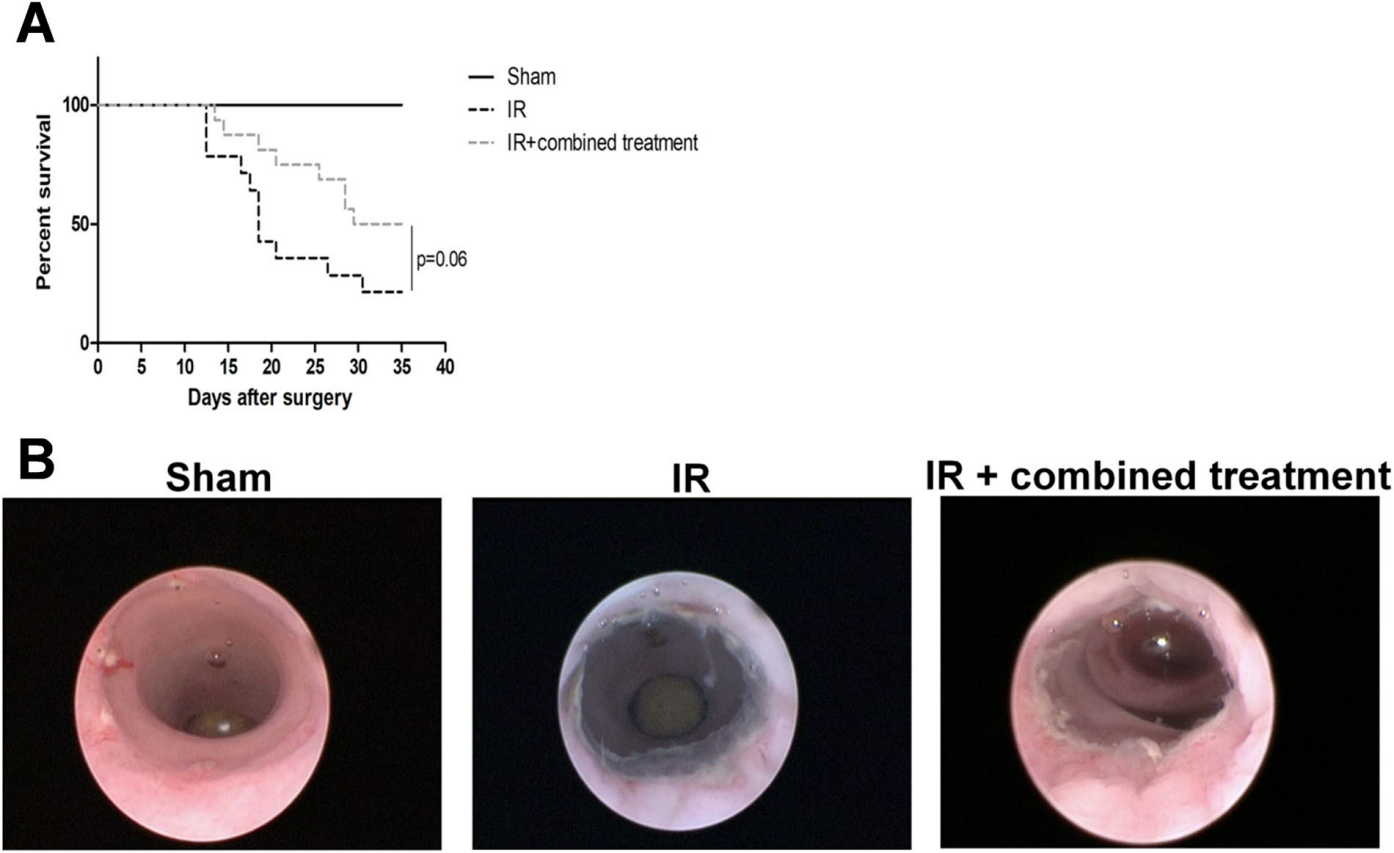
Improvement of animal survival following colonic anastomosis after irradiation using combined treatment (iterative injections). Survival curves were compared with the Fine and Gray competing risk regression model using R (R foundation). The competitive risks methodology was used to discriminate non-surgery-related deaths (during MSC injection) and surgery-induced mortality. Representative pictures of colonoscopy in the different groups. N = 16 animals per group. Sham = control rats with surgery, IR = irradiated rats with surgery, IR + combined treatment = irradiated rat + surgery + combined treatment injected as described in the protocol 2 of Supplementary Fig. 2.

A first set of experiments was performed to design and develop the procedure of surgery after irradiation. The colonic anastomosis was performed in control and irradiated rats. The distal colon was cut in the middle of the irradiation zone (white and non-vascularized area) and reconnected. In control rats the same procedure was applied. Using this method, all the irradiated rats died between 5 and 14 days after the surgery while the control rats survived. (They were euthanized 4 weeks after the surgery). Thus, we cut the distal colon just above the irradiated area before anastomosis and all animals (irradiated or not) survived. Then, in the irradiated groups, the colon was cut in the middle of the irradiated area on one side and above the irradiation area (visualized as vascularized zone) on the other side. The anastomosis was performed between a normal and an irradiated area (data not shown). Using this procedure of surgery, we obtained 100% of survival in the sham group and 33% in the irradiated group (Fig. 6A). Thus, the latter method was used to study the therapeutic benefit of the combined treatment to improve the healing of colonic anastomoses after irradiation.

### Combined treatment improves colonic anastomosis healing following irradiation

The protocol of the combined treatment on surgery procedure is summarized in the Supplementary Fig. 2. We observed that the combined treatment on irradiated rats induced a shift of the survival curve (p = 0.06) compared with irradiated and non-treated animals (Fig. 6A). Eight weeks after irradiation (5 weeks after surgery), the colonic mucosa was observed using colonoscopy (Fig. 6B and representative videos were shown in supplementary data/online). We observed a complete healing of colonic anastomosis in the sham group. After irradiation and surgery procedure, the colonic anastomosis showed a lot of necrotic tissues and fibrin as viewed by white deposits. In the irradiated and treated group, we observed fewer necrotic patches around the anastomosis compared to irradiated rats. The rat autopsy showed no perforation, but abundant fibrotic tissue located in the surgery zone in irradiated and non-treated rats (data not shown).

### Assessment of the therapeutic benefit using histological parameters

**Figure 7:**
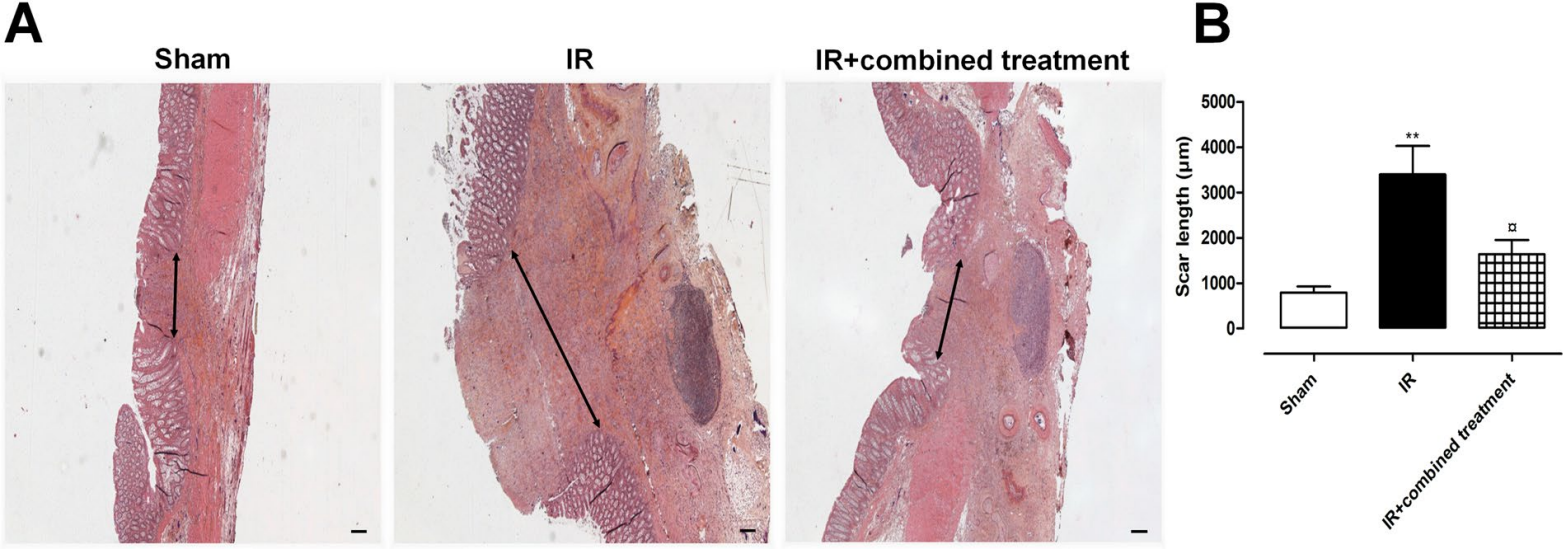
Improvement of colonic anastomosis healing after irradiation using combined treatment (iterative injections). (**A**) Representative pictures of colonic damage visualized by HES staining 5 weeks after surgery, thus 8 weeks after irradiation in the different groups of rats. Scale bars represent 200 µm. (**B**) Measurement of the scar length 8 weeks after irradiation. N = 5 animals per group. Sham = control rats with surgery, IR = irradiated rats with surgery, IR + combined treatment = irradiated rat + surgery + combined treatment injected as described in the protocol 2 of Supplementary Fig. 2. ^**^p < 0.01 vs. sham rats, ^¤^p < 0.05 vs. IR.

**Figure 8:**
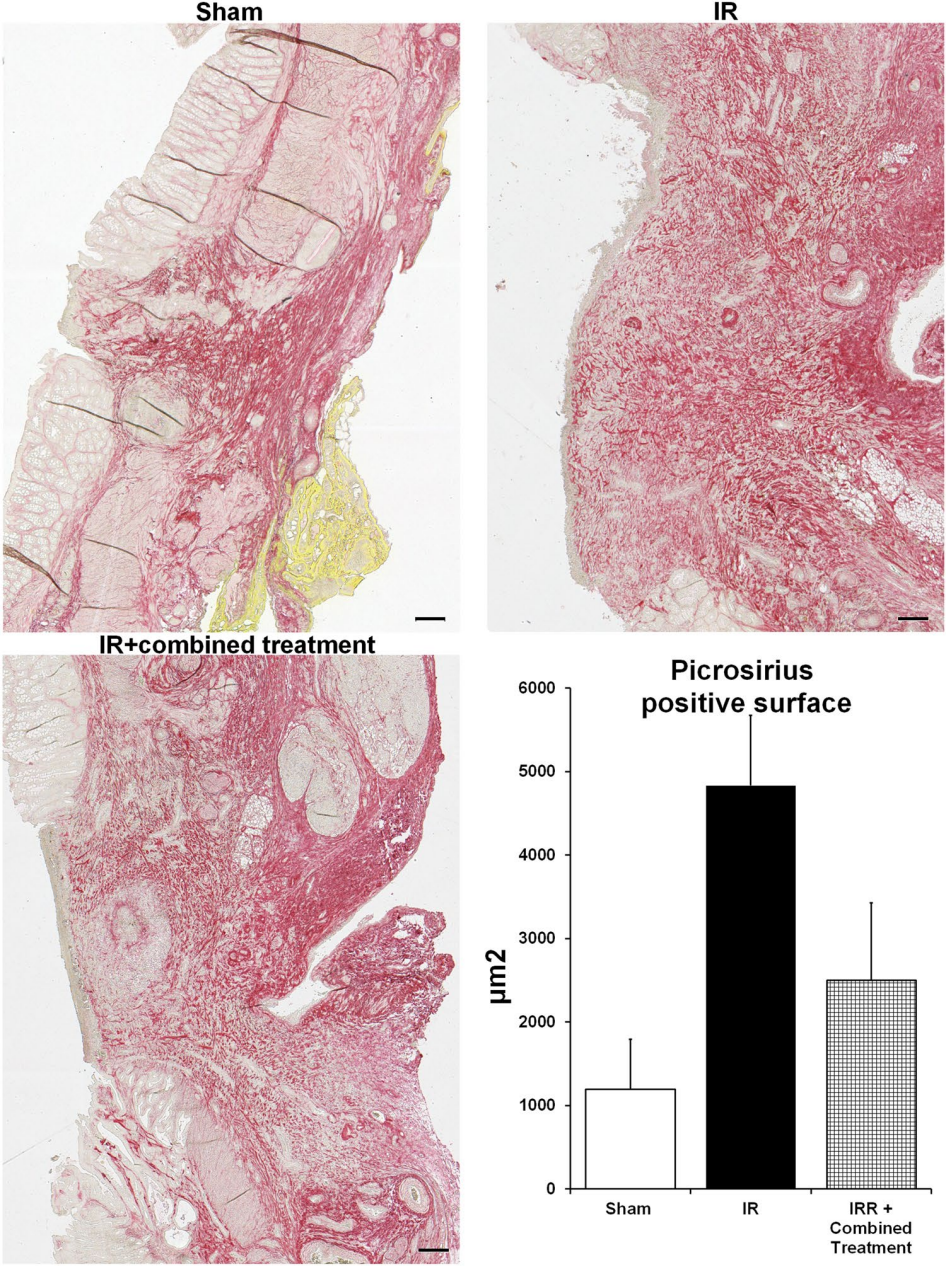
Study of collagen deposit following colonic anastomosis after irradiation using combined treatment (iterative injections). (**A**) Representative pictures of picrosirius staining 5 weeks after surgery thus 8 weeks after irradiation in the different groups of rats. Scale bars represent 200 µm. (**B**) Quantification of the ratio of the surface picrosirius positive staining to the total surface of the anastomosis scar. N = 5 animals per group Sham = control rat with surgery, IR = irradiated rat with surgery, IR + combined treatment = irradiated rat + surgery + combined treatment injected as described in the protocol 2 of Supplementary Fig. 2. ^**^p < 0.01 vs. sham rats.

The anastomotic healing process was assessed by measuring the size of the colonic scar (area without crypt; ulcer) (Fig. 7A). We observed that the size of the scar was statistically reduced in irradiated treated animals compared with irradiated non-treated rats (Fig. 7B). Picrosirius red staining is a commonly used histological technique to visualize collagen fibers. We quantified the surface of Picrosirius positive staining after colonic anastomosis in sham, irradiated and irradiated-treated rats. We observed that the surface of red staining increased after irradiation and surgery compared to controls (Fig. 8). A slight decrease of Picrosirius positive collagen surface was observed at the scar after the combined treatment (Fig. 8).

## Discussion

Damage of healthy tissues surrounding pelvic tumors remains unavoidable so far. This disease severely affects the quality of life of patients, due to some refractory lesions such as ulcer, chronic inflammation, or hemorrhage which could progress to fatal complications such as obstruction, fistulae or perforation. Current prevailing therapies could in some cases alleviate the symptoms, but their efficacies are still not evidenced on severe damage. In recent years, preclinical studies then clinical trials demonstrated the therapeutic benefit of cell therapy using MSCs in various pathologies. Based on hundreds of clinical trials, the safety of this therapy appears clear^18^, however the results are more elusive concerning the clinical efficacy^19^. There is still much to learn about MSC action mechanisms and optimization of the protocol of treatment with regard to the pathology to be treated. It is generally considered that the reparative properties of MSCs are exerted by secretion of a wide variety of bioactive molecules that could operate directly via interaction with cells in wounded areas or indirectly by the stimulation of endogenous host progenitor cells^20–22^. As few MSC engraftment has been demonstrated, various strategies aimed to increase the number or the biologic action of MSCs in the tissue. Interestingly, these studies demonstrated a correlation between the presence of the cells and the therapeutic benefits (for review^1^). Indeed, in our lab, we previously demonstrated that a local injection of MSCs within a Si-HPMC hydrogel increased MSC engraftment associated with the improvement of the colonic function and structure in the margin zones after irradiation. However, in the case of extended colonic lesion, the ulcerated area persists. Thus, in this study, we tested a new concept that is clinically translatable. The objective of this new strategy is not just to increase MSC engraftment in the tissue but also to improve the efficacy of MSC-secreted factors with an action on the ECM damage after irradiation, using a HS-m: RGTA^®^ molecule. RGTA^®^ is already used in clinic in two commercially available products, one to treat chronic skin lesions^23^ and the other for corneal lesions^24^. In injured tissue, and more precisely after irradiation inducing reactive oxygen species (ROS) and huge neutrophils and mastocytes infiltrates^9^, HS present at the cell surface and within the ECM are degraded by enzymes. Thus, free HS binding sites are limited to wounded tissue. Previous study demonstrated that RGTA^®^ could be detected in the injured muscle at least one week after systemic administration^25^. RGTA^®^ is a non-acting molecule and its application should be timed, indeed excess of RGTA^®^ could compete for heparan binding growth factors. As previously described, in this study we demonstrated that weekly administration, through intravenous route, seems appropriate to induce therapeutic benefit^16,26^.

Irradiation-induced damages are very complicated lesions because they combine cell depletion with a destruction of the stem-cell compartment associated with a chronic inflammatory process and an activation of the immune system. In addition, intestinal permeability is a functional feature of the intestinal barrier and represents a pathological feature of patients treated with radiotherapy^27^. An intact intestinal barrier prevents the entry of antigens, endotoxins and pathogens into the organism, whereas intestinal dis-integrity allows their entry, which may initiate inflammation and disease^28^. In our model, an increase in intestinal permeability to FD4 and HRP was observed 4 weeks after irradiation. A loss of the epithelial integrity after irradiation was supported by our results of increased permeability to FD4 because of the size of this molecule which allows the use of the paracellular route^29^. The increased permeability to HRP, which normally does not permeate or permeates at a low level, indicates severe damage to the epithelium allowing transcellular passage to macromolecules and then to bacteria^30^. The risk of septicaemia is thus evident. In this study, we tested a new combination to treat this complex pathology. MSCs act through secretion of various molecules (anti-apoptotic, anti-inflammatory, chemokines for progenitors’ recruitment, pro-regenerative (pro-angiogenic and pro-proliferative)) that could modify various tissue compartments. The fact that MSCs act at various levels means that these cells remain a good candidate to improve wound healing after irradiation. Local injection of MSCs embedded in hydrogel induces cell engraftment in irradiated colonic mucosa until 21 days^14^ and co-treatment with HS-m didn’t further increase MSC engraftment (Supplementary Fig. 5). The therapeutic benefit seems to reach a plateau. Thus, in this study, we aimed to condition the host with an HS-mimetic to prepare the microenvironment before MSCs injection. Using this combined procedure, we demonstrated a statistical improvement of the functional permeability parameters. Interestingly, besides the efficiency on the margin areas detected by histology, we highlighted a reduction of the lesion score in the ulcerated area. This therapeutic benefit could be explained by an involvement of the HS-mimetic at different levels. The ability of the HS-mimetic to participate in the architectural reconstruction of the ECM scaffold is the first necessary step in reestablishing a microenvironment conducive to tissue repair. In the case of colonic mucosa, the stem cells are located at the bottom of the crypt. The crypt is surrounded by pericryptal cells known as the stem cell niche that generates signals involved in stem-cell maintenance and control of cell proliferation and differentiation. The HS-mimetic, by restoring the niche, could favor signals to induce cell proliferation following injury. Although no specific receptors for HS-mimetic are known, it possibly interacts with diverse HS binding proteins. It has been demonstrated to interact with Stromal cell-Derived Factor 1α (SDF1α) chemoattractant, growth factors such as Fibroblast growth factor (FGF), Vascular endothelial growth factor (VEGF), and anti-inflammatory cytokines such as Transforming Growth Factor β (TGFβ) (revue^15^). Specifically, these molecules have been demonstrated to be secreted by MSCs^12^ and could reinforce the recruitment of endogenous MSCs^7^, and the pro-regenerative and anti-inflammatory action of MSCs^31^. It has been demonstrated that HS-m treatment has no effect on gene transcription, however protein content is increased^32^. These observations strengthen the role of HS-m in sequestrating/stabilizing/protecting proteins rather than increasing the capacity of gene expression. We realized *in vitro* analysis on MSC and, in the same way, we demonstrated a statistical increase of pro-regenerating factors content in the culture supernatant (VEGF, FGF, granulocyte macrophage colony-stimulating factor; (G-CSF), and Platelet derived growth factor (PDGF)) in presence of HS-m (Supplementary Fig. 6). Moreover, proteins that bind HS provide better activity than their soluble forms^33^. We previously demonstrated that interaction of glycosaminoglycan (GAG)-mimetics with TGFβ increase the biological effect of this osteochondrogenic-inducing factor^34^. Recently, paracrine action of MSCs also includes production of extracellular vesicles (EV) that carry nucleic acids and proteins. The production of EV by cells is involved in cell communication with the ability to modify the activity of the target cell. The uptake of EV by the recipient cell is a crucial step that could be modulated by HS^35^. Several intracellular signaling pathways, such as the developmentally conserved wingless integration site (wnt)/β-catenin pathways, have critical roles in crypt-villous homeostasis^36^ and regenerative process after injury. Interestingly, it has been demonstrated that HS increase Wnt-binding affinity, promote the phosphorylation of Wnt coreceptor LRP6 (Low-density lipoprotein receptor-related protein-6) and enhance Wnt/β-catenin signaling^37^. We previously reported that supernatant of MSC promoted β-catenin nuclear localization and increased the number of epithelial cells after irradiation. Moreover, MSC treatment following colonic irradiation increased Wnt4 expression^7^. In this study, we demonstrated *in vivo* an increase of crypt epithelial proliferation that could explain the ulcer healing after the combined protocol. Indeed, intestinal mucosa regenerates through healthy margins boarding the ulcer. Altogether, we could hypothesize that short-term after-MSC local injection, MSC-secreted factors bind to HS-m, increasing their life span and their ability to induce functional effect to reinforce the regenerative potential of the on-host tissue.

These encouraging results give us good reasons to test this new combination to improve healing of colonic anastomosis after irradiation. Anastomotic dehiscence is one of the most serious potential complications after colorectal surgery in patients. Tumor reduction with pre-operative radiotherapy is considered to increase the risk of esophageal, colonic and rectal anastomotic leakage^38–41^. Other factors favoring anastomotic leakage have been identified including patient predisposition and the use of certain drugs like steroids. Several studies using tissue adhesive are ongoing, however, further investigations are needed before their use in clinic. Nonoperative management with pharmacological interventions is limited^42^. The use of preventive antibiotic perioperatively has become a standard care. As the colon harbors the densest bacterial population, surgery procedure is associated with a high rate of infection compared to other tissues. The presence of foreign antigens can lead to a huge inflammatory response marked by the presence of innate immune cell releasing a high number of proteases and ROS that could participate in ECM damage followed by anastomotic leakage.

Anastomotic healing is a dynamic process that flows on through multiple phases beginning immediately after the apposition^43^. Generally, at the site of injury, a vasoconstriction and an activation of the clotting cascade occurs. Within the clot itself, the conversion of fibrinogen to fibrin constitutes the initial step of the construction of the provisional ECM. This scaffold functions by facilitating inflammatory and mesenchymal cell migration. The presence of thrombin and platelets favors this process, in particular through releasing factors. Then follows the inflammatory phase with increased vascular permeability and inflammatory cell infiltrate. The inflammatory cells secrete molecules that modulate for instance angiogenesis, re-epithelization and collagen deposition. During the subsequent proliferative phase, collagen and ECM are synthetized. The cellularity of the wound increases due to the migration and proliferation of endothelial, epithelial, smooth muscle and fibroblast cells within the ECM, associated with an increase of growth factors secreted by these cells. Finally, a maturation phase occurs, collagen homeostasis is achieved, which is marked by changes in collagen types as well as an increase in tensile strength through a cross linking of collagen fiber.

Due to the importance of ECM on anastomotic healing, we tested our new combination to favor colonic anastomosis healing after irradiation. Our results determined an improvement in rat survival after the treatment. Using this protocol, we could evaluate the therapeutic effect of the combined treatment on ECM remodelling over a longer term. Collagen synthesis is necessary in the early stage of wound healing; however, an over-deposition of collagen could result in scarring. In this study, we observed an increase of collagen deposit measured in the scar of the anastomosis after irradiation. The combined treatment induces a decrease in collagen deposit suggesting a better healing process. Indeed, it has been previously demonstrated that RGTA^®^ reduces overexpression of collagen 3 and normalizes the ratio collagen 1 to collagen 3 *in vitro*^44,45^ and *in vivo* in skin^46,47^ and in normal and irradiated intestine tissues^48^ including biopsies from patients with Crohn disease^49^. Hydrogels were used because of their structural and compositional resemblance to the natural ECM. These materials can be designed to manage cellular attachment, molecular responses, structural integrity, biodegradability and biocompatibity^50^. The Si-HPMC hydrogel composition (1.5% w/w) used in this study could allow cell migration and enables diffusion of factors secreted by embedded MSC to exert biological influences and modify cell behaviour. As previously demonstrated, Si-HPMC encapsulated MSCs could be retrieved in the colon 7 days after injection; the quantity then decreased and few cells were retrieved in the colon 21 days after injection^14^. Thus, conditioning the host with an HS-m could favour the efficacy of wound healing, driven by MSC secreted factors, reversing the hostile microenvironment induced after irradiation plus surgery, and fostering tissue healing.

## Conclusions

Late side effects induced after irradiation become an increasing clinical problem. In this study, we propose a new procedure of treatment that combines cell therapy, biomaterials and heparan sulfate mimetics. We demonstrated, for the first time, a reduction of the lesional score in the ulcerated zone of the colon in a relevant rat model of the human pathology. Moreover, in a model of colonic anastomosis following irradiation, we observed an improvement of animal survival and scar healing. The therapeutic benefit of this new approach proposed herein, in two different models, opens up new prospects optimizing cell therapy and can be proposed to patients suffering from severe colonic defect after radiotherapy.

## Materials and Methods

### Animals and irradiation protocol

Male SD (Sprague Dawley) rats (Janvier SA, Le Genest St Isle, France) weighing 250 g were housed in a temperature-controlled room (21 ± 1 °C). Rats were exposed to a 12-hour light/dark cycle and fed with standard pellets with food and water available ad libitum. Rats were anesthetized by isoflurane inhalation. A single 29 Gray (Gy) dose was delivered by a medical accelerator through a 2 × 3 cm window centered on the colorectal region. The accelerator has a maximal energy of 4 MeV with an average energy of about 1.5 MeV; 30 kA.

This study was carried out in strict accordance with the recommendations of the Guide for the Care and Use of Laboratory Animals as published by the French regulations for animal experiments of the Ministry of Agriculture with European Directives (86/609/CEE). Animal facilities of the Institute have the agreement number C92-032-01. The experimental protocol was reviewed by The Ethical Committee of the Institute that is registered by the “Comité national de réflexion éthique sur l’expérimentation animale” under the number 81. The experimental protocol (P13-14) was submitted to the French national authorization platform and after approval was registered under the permit number #6247-2016072910589302.

### Cell culture and characterization

Adipose-derived MSCs were obtained by digesting the subcutaneous inguinal adipose tissue of seven-week-old GFP-transgenic SD rats as previously described^10^. The phenotype of amplified Ad-MSCs was verified by flow cytometry. The percentage of CD90 (clone OX-7; BD Biosciences, Le pont de Claix, France) and CD73 (clone 5F/B9; BD Biosciences) positive cells were analyzed and the absence of hematopoietic cells was verified with CD34 (clone ICO115, Santa Cruz, Dallas, Texas, USA) and CD45 (clone OX-1; BD Biosciences) markers. Isotype identical antibodies served as controls. The potential of adipogenic and osteogenic differentiations was also verified before injection. Cells were plated at 10 000 cells per cm^2^, at 80% of confluence, inductive media were applied during 3 weeks. For osteogenic differentiation: MEMα (Minimum Essential Medium) 10% FCS (foetal calf serum), 10 mM β–glycerophosphate, 10^−7^ M Dexamethasone, 50 µg/ml L-ascorbic Acid. For adipogenic differentiation: MEMα 10% SVF, 100 µM Isobutyl methylxanthine, 10^−7^ M Dexamethanone, 60 µM Indomethacin, 10 µg/ml Insulin.

### Hydrogel preparation

Hydroxypropyl methyl cellulose (Methocel^®^ E4M Premium procured from Colorcon) was silanized by grafting 3-glycidoxypropyltrimethoxysilane GPTMS as previously described^51^. Si-HPMC powder was dissolved in NaOH solution (0.2 M) then dialyzed to reach a final pH value of 12.7^52^. Acidic buffer solution used to neutralize the basic Si-HPMC solution was prepared using 0.1 M HCl and HEPES as previously described^52^. Si-HPMC hydrogels were prepared by rapidly mixing the polymer and the buffer at a ratio of 2:1 leading to a pH of 7.4 and then, initiating silanol condensation.

### RGTA^®^ preparation

RGTA^®^ was obtained from OTR3 Company as an injectable GMP quality substance to dissolve in saline. RGTA^®^ is an alpha1-6 polyglucose carboxylmethyl sulfate derivative prepared from 40kD alpha1-6 polyglucose by carboxymethylation with monochloroacetic acid treatment, followed by O-sulfation with SO_3_-DMF complex in the presence of 2-methyl-2-butene (Papy *et al*. Macromolecules 2005).

### Experimental protocols

A scheme of the two procedures used in this study is provided in a Supplementary Figure. In two series of experiments, 3 weeks after irradiation, rats were injected with RGTA^®^ intravenously (IV) or MSCs locally injected with Si-HPMC using a colonoscope (Karl Storz - Endoskope, Tuttlingen, Germany) or a combination of both these treatments. These groups are called HS-m, MSC-Hy and MSC-Hy + HS-m respectively in the figures. For IV injection, RGTA^®^ (1 mg/kg) was diluted in 500 µl of PBS and injected in the tail vein. For local injection, 1.10^6^ of MSCs from adipose tissue was mixed with 300 µl of Si-HPMC (1.5%) and injected locally using the colonoscope. An injector of 18 cm in length and 1.5 mm in diameter connected to a needle (21Gauge) was used. Two injection points were made, upstream and downstream of the lesion. Animals were then sacrificed one week later. In the first series, distal colons were dissected, washed in Krebs solution and used for colonic epithelial permeability measurements in Ussing chambers. A second series of experiments was performed where samples of colon were collected for histological and immunohistochemistry staining.

In a third series of experiments, a colorectal anastomosis was performed three weeks after irradiation. One day before the surgery, RGTA^®^ (1 mg/kg) IV was injected in the tail vein. During the surgery, treated rats were injected with a combination of 1.10^6^ MSCs locally injected with Si-HPMC at 1.5% of concentration. Then, three intravenous injections of 1.10^6^ MSCs were realized every 10 days and six intravenous injections of RGTA^®^ every week. Previous experiments, using MSC injections alone demonstrated no increase of rats’ survival (data not shown). For ethical reasons (protocol P13-14), this group was not included in the experiment of the present study. Only groups with treatments similar to those that would be used in clinical compassionate case were tested. Colonoscopy was performed before euthanasia. Rats were sacrificed 8 weeks after irradiation.

### Ussing chambers experiments

Immediately after the euthanasia of the rats, distal colons were cut along the mesenteric border, and three colonic strips from each rat were used to assess colonic permeability using Ussing chambers (Corning Costar Corporation, Harvard Apparatus, France) with a flux area of 0.1 cm^2^. Colonic epithelial permeability to small and large molecules was assessed by measuring mucosal-to-serosal passage of fluoro isothiocyanate (FITC)-Dextran 4 kDa (FD4; 2.2 mg/ml) and intact Horseradish peroxidase (HRP; 0.4 mg/ml) 44 kDa (Sigma-Aldrich, Saint Quentin Fallavier, France) during 2 hours. Colonic permeability to FD4 was determined by measuring the fluorescence intensity at 485 nm/525 nm using an automatic Infinite M200 microplate reader (Tecan, Lyon, France). Epithelial permeability to intact HRP was determined by an enzymatic assay^53^ for specific HRP activity found in the serosal and mucosal compartment using a microplate reader (Tecan). Permeability was calculated as the ratio of flux/concentration, as previously described^54^ and expressed in cm/second. Data are the means of triplicate measurements.

### Analyzes of the colonic lesions and histological studies

Immediately after the sacrifice, pictures of the colonic pieces were taken and the irradiated area corresponding to a white and non-vascularized zone was measured to evaluate the macroscopic damage. Then, the colon was collected and fixed in 4% formaldehyde and embedded in paraffin. Paraffin embedded colons were cut on a rotary microtome (Leica Microsystems AG, Wetzlar, Germany) into serial circular sections of 5 µm, spaced 150 µm and stained with hematoxylin-eosin-saffron (HES). The severity of colorectal damage induced by irradiation was assessed using an injury score. Determination of the injury score was based on mucosal damage (ulceration, epithelial atypia, and regenerative crypts), edema, colitis cystica profunda, vascular sclerosis and muscular dystrophy. Graduation of the injury was 0 = null; 10 = slight; 20 = moderate and 30 = severe. The score was realized along the damaged zone which is around 50 slides. The mean score was determined according to the zone as determined in the Fig. 1. Composition in collagens was evaluated using Picrosirius on longitudinal slides of the colon after surgery. Histolab software (Microvision Instruments, Lisses, France) was used to automatically detect Picrosirius stained surfaces. Quantification of collagen deposition is obtained by dividing the Picrosirius stained surfaces area by the total surface of surgery zone, which was manually measured.

### Immunohistochemistry

For immunohistochemistry (IHC), sections were deparaffinized and hydrated. Serial slides were used for PCNA (Proliferating Cell Nuclear Antigen) staining. Tissue sections were treated with 0.1% triton X-100 (Sigma-Aldrich) in PBS 1x (Life Technologies) at room temperature (RT) for 10 min. Then endogenous peroxidases were inhibited by incubation with 3%H_2_O_2_ in methanol at RT for 10 min. Tissue sections were then placed in an antigen retrieval solution (0.01 M citrate buffer, pH = 6 (Zytomed, Berlin, Germany) for 15 min at 350 W) and quenched for endogenous peroxidases as described above. After saturation (GBI labs, Washington, USA), mouse anti-PCNA (M0879, DakoCytomation, Courtaboeuf, France) at 525 µg/ml was applied for 1 h at 37 °C. Sections were incubated with a kit anti-mouse HRP (GBI labs) for 30 min at RT. Staining was developed with HRP Green (Zytomed). Then, sections were counterstained with nuclear fast red (H3403, VectorLabs, Burlingame, CA, USA), dehydrated and mounted. Isotype control antibodies are used as negative controls.

### Surgery

Surgery was performed under general anesthesia induced with isoflurane 5%. In sham rats, a part of the distal colon was cut and removed. In the irradiated groups, the colon was cut in the center of the irradiated area (white and non-vascularized area) and above the irradiation, in a normal vascularized zone in order to connect a normal with an irradiated area. The colon was connected end to end with separate poliglecaprone stitches (Monocryl® 6/0, Ethicon, Issy-les-Moulineaux, France) with all the knots outside. MSC-Hy or PBS were injected locally after the surgery. The abdomen was closed with polyglactin 910 (Vicryl® 4/0, Ethicon) running suture and the skin with polyglactin 910 (Vicryl® 3/0, Ethicon) horizontal mattress suture. A pain killer (Buprecare^®^ 0.3 mg/ml, Axience, Pantin, France) was subcutaneously injected. Animal survival was monitored every day during the 8 weeks of the experiment.

### Colonoscopy

Videos for colonic anastomosis healing were performed at 8 weeks after irradiation on anesthetized rats with a colonoscope (Karl Storz, Germany).

### Statistical analysis

All data are presented as mean ± SEM. Statistical analyzes were performed using Graph Pad Prism 5.0 (GraphPad, San Diego, CA). Statistical significance was determined using t-test for a comparison between two groups, one-way ANOVA followed by Tukey’s post-test for multiple groups, or two-way ANOVA followed by Bonferroni’s post-test for multiple groups with different variables. Survival curves were compared with the Fine and Gray competing risk regression model using R (R foundation). The competitive risks methodology was used to discriminate non-surgery-related deaths (during MSC injection) and surgery-induced mortality. Statistical significance was set at p < 0.05.

## Electronic supplementary material

Supplementary figures
