# Supplementary material for: Heparan Sulfate Mimetics: A New Way to Optimize Therapeutic Effects of Hydrogel-Embedded Mesenchymal Stromal Cells in Colonic Radiation-Induced Damage
Source: Sci Rep. 2019 Jan 17;9:164. doi: 10.1038/s41598-018-36631-6 (PMC6336771; doi:10.1038/s41598-018-36631-6)
Supplement: Supplementary file 1 — Supplementary figures [file 41598_2018_36631_MOESM1_ESM.docx]

**Heparan Sulfate Mimetics: A New Way to Optimize Therapeutic Effects of Hydrogel-Embedded Mesenchymal Stromal Cells**

**in Colonic Radiation-Induced Damage**

Lara Moussa, Christelle Demarquay, Gildas Réthoré, Mohamedamine Benadjaoud, Fernando Siñeriz, Girish Pattapa,Jérôme Guicheux, Pierre Weiss, Denis Barritault, Noëlle Mathieu^*^

**Supplementary figures**

***Supplementary Figure 1: Histology and permeability analyses at 2, 4 and 8 weeks after irradiation.***

*Representative pictures of colonic damage induced after localized irradiation using HES staining (upper panel). Measurement of colonic permeability to FD4 using Ussing Chambers. ^**^p<0.01 vs. control rats (lower panel).*


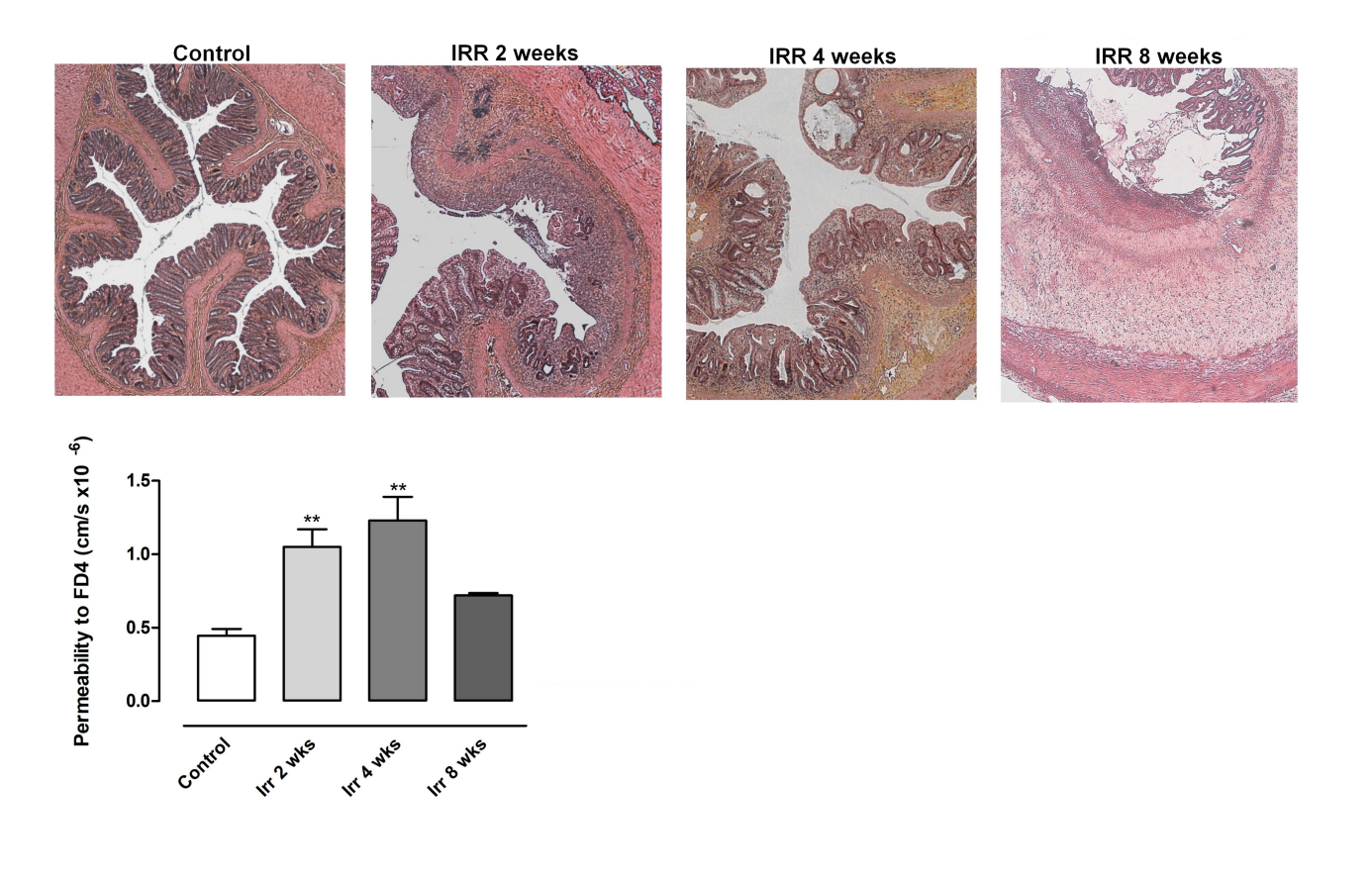


***Supplementary Figure 2: Schematic representation of the 2 procedures used in this study.***


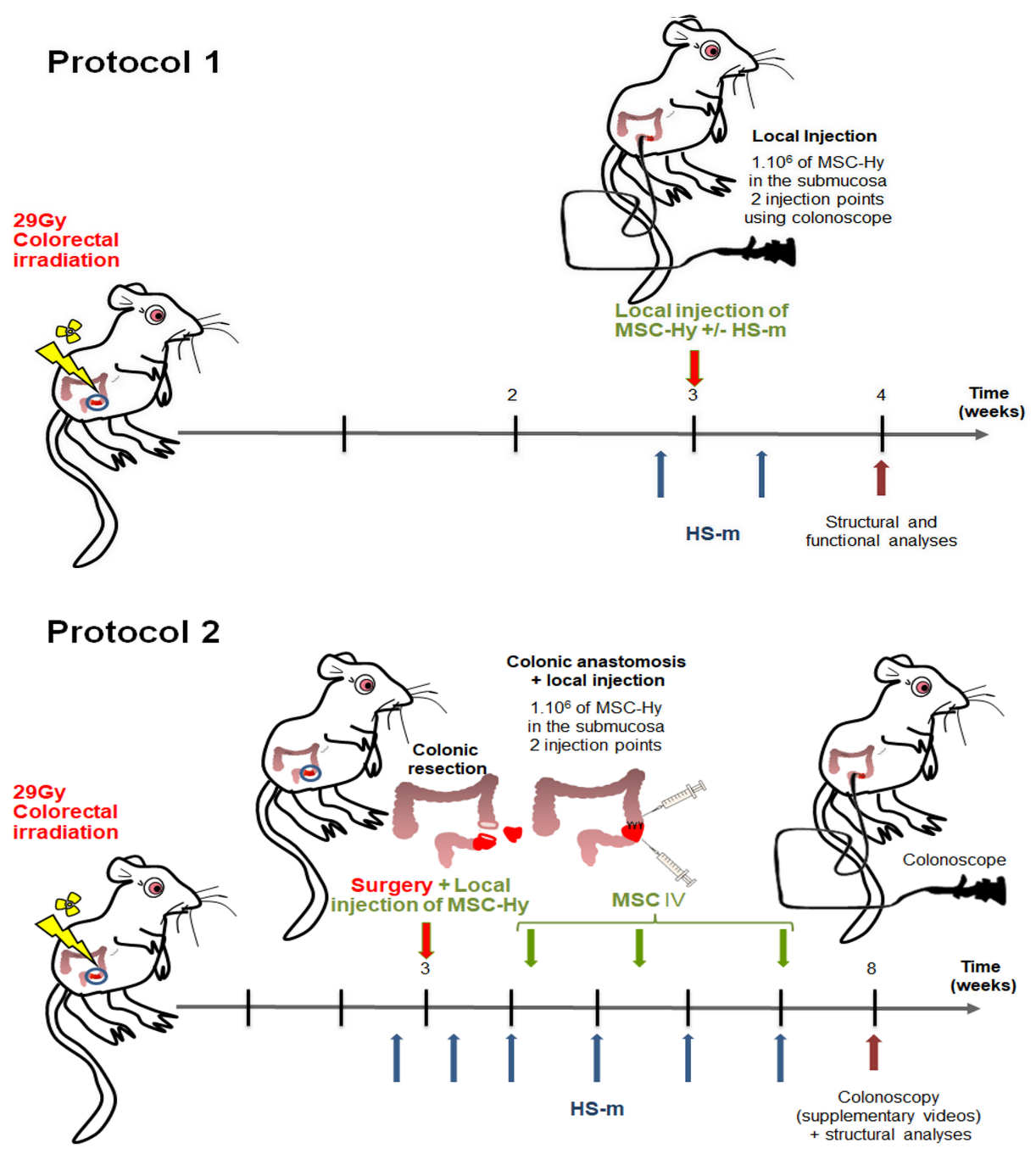


*In Protocol 1, the rats were irradiated through a colorectal window and experiments lasted 4 weeks.*

*Injection of the HS-m: RGTA*^®^ *(1mg/kg) was injected intravenously in the tail vein of the rats as mentioned by the blue arrows (2 injections separated by 4 days) or RGTA® (100µg/ml) was locally injected with MSC-hy by colonoscopy 3 weeks after irradiation.*

*Injection of MSC: 1 million MSCs from adipose tissue were embedded within Si-HPMC hydrogel at 1.5% and injected locally using the colonoscope in two injection points in the colonic mucosa. MSCs were injected 3 weeks after irradiation as mentioned by the red arrow and structural / functional analyses were realized 4 weeks after irradiation.*

*For the combined treatment the 2 procedures were mixed respecting the kinetic.*

*Protocol 2 demonstrated the procedure of colonic anastomosis and combined treatment. Three weeks after irradiation, the surgery was performed by a resection of a part of the irradiated colon (red) and a reconnection of the rest of the irradiated colon with a normal zone (pink). Injection of MSC: An injection of one million MSCs from adipose tissue with 1.5% Si-HPMC locally was done at the time of the surgery (as indicated by the syringes). One million MSCs from adipose tissue were then injected intravenously every 10 days (Total of 3 injections).*

*Injection of the HS-m: RGTA*^®^ *(1mg/kg) was injected intravenously in the tail vein of the rats as mentioned by the blue arrows (total of 6 injections separated almost each week).*

*For the combined treatment, the 2 procedures were mixed respecting the kinetic*

*Eight weeks after irradiation (5 weeks after surgery), a colonoscopy was performed (videos in supplementary data) and the surviving animals were sacrificed for histological analyses.*

***Supplementary Figure 3: Characterization of MSC phenotype before injection***

*Flow cytometry analysis of MSC before injection; the absence of hematopoietic markers (in red) and the presence of mesenchymal markers (in blue) was verified. We also checked the expression of the GFP protein. The table represents expression of five different MSC preparation from GFP-SD rats.*


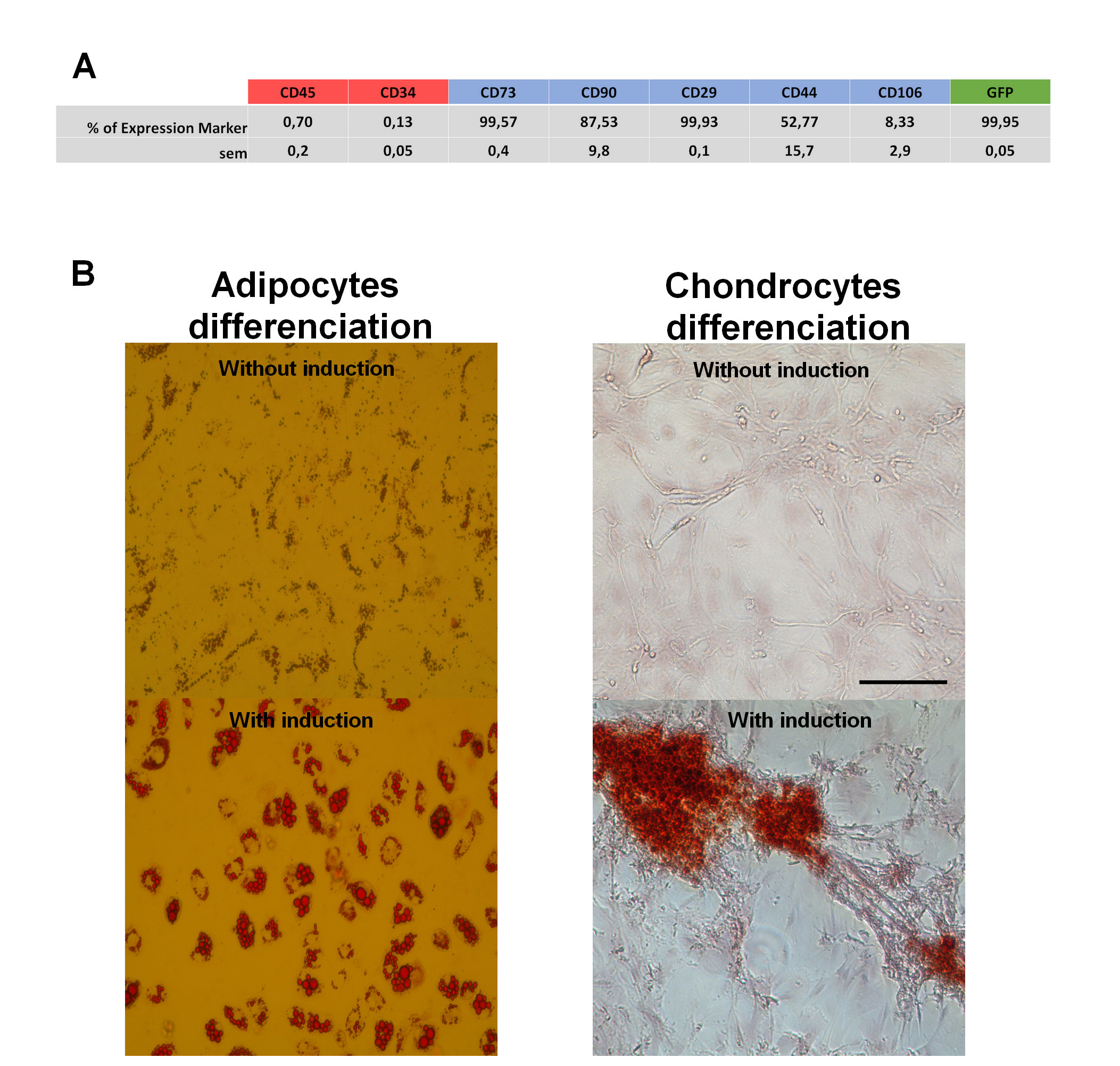


***Supplementary Figure 4: Viability and activity of MSC in presence of HS-m***


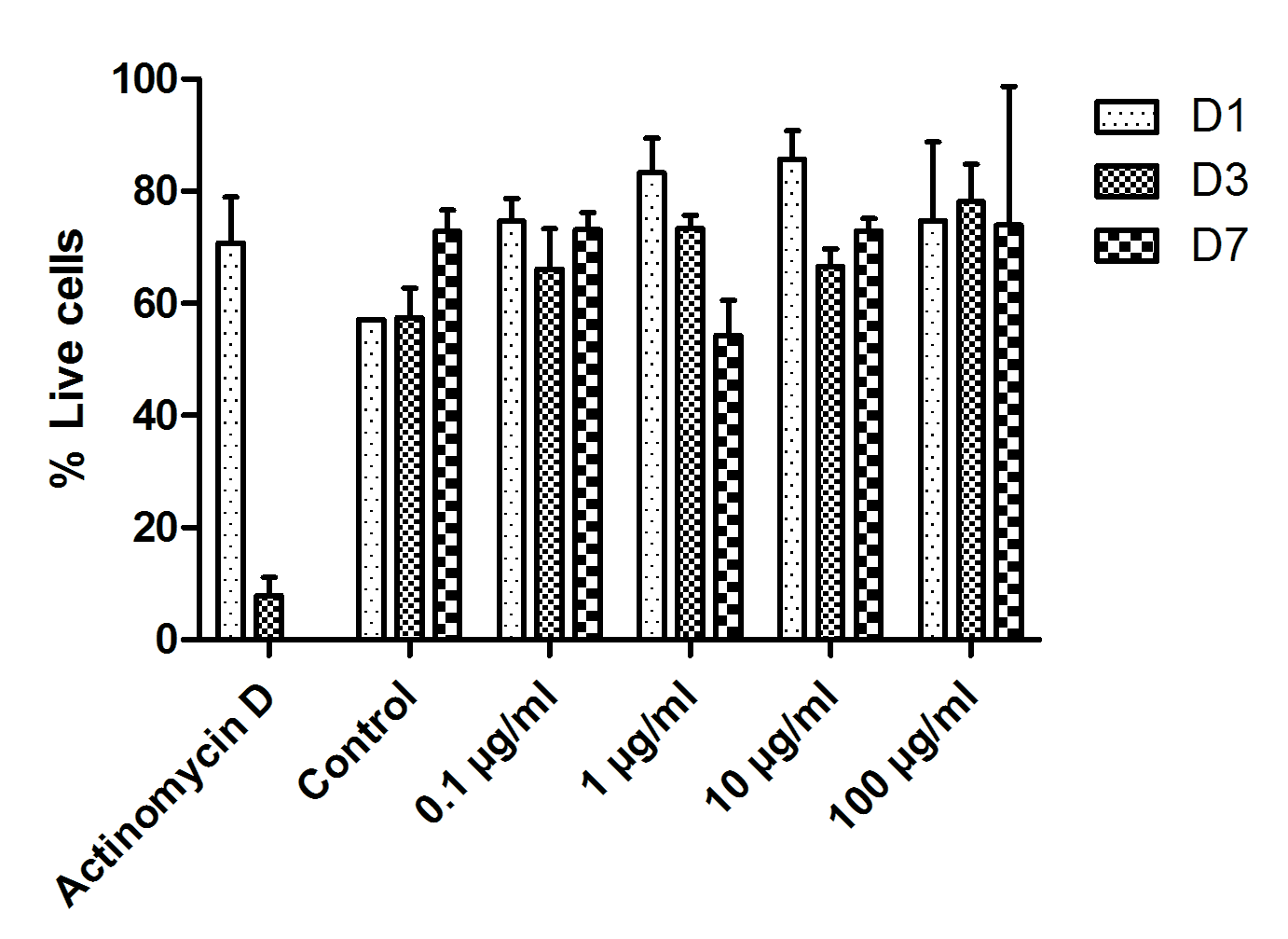


**A**

*A- Viability of MSC-hy is not modified by increasing concentrations of HS-m (0.1 to 100µg/ml). Cell viability was evaluated by Live/Dead Cell Viability assay according to the manufacturer’s instructions (ThermoFisher Scientific, MA, USA) in MSC mixed into Hy at a final density of 1.10 6 cells per mL of Hy. Hy containing different concentrations of HS-m were molded and allowed to gelate in wells of a 48-well plate at 37°C for 1h. After gelation, 200 µL of culture medium were added to each well and refreshed every 2 days. As positive control, MSCs were cultured in Hy in the presence of actinomycin-D (5 µg/mL) a well-known inducer of cell death. Living cells were stained green and dead cells were stained red. Red and green fluorescence were observed with a confocal microscope (Nikon D-eclipse C1 (Ar/Kr)). Each condition was tested in quadruplicate.*


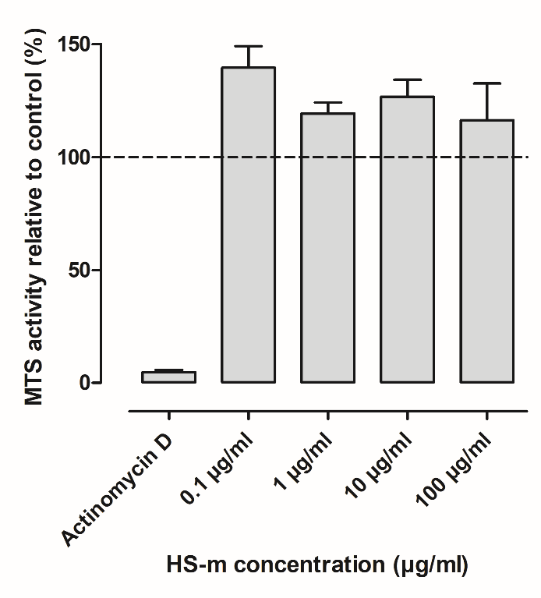


**B**

*B- Mitochondrial activity of MSC is not statistically modified by increasing concentrations of HS-m (0.1 to 100µg/ml). MSC activity was evaluated by methyl tetrazolium salt (MTS) assay (Promega, USA). MSCs were seeded onto culture plates and allowed to attach to 48-well plates at a final density of 10,000 cells per cm². After 24h, the culture medium was removed and Hy containing HS-m was added onto the cell layer. After 1h of gelation at 37°C, 200 µL of culture medium was added to each well and refreshed every 2 days. As a positive*

*control, MSCs were cultured in the presence of actinomycin-D (5 µg/mL). The MTS assay is based on the reduction of MTS tetrazolium compound by viable cells that generates a colored formazan product soluble in culture medium. The colored product was measured by the optical density reading at 490 nm (Victor 3 V 1420 Multilabel Counter). Each condition was tested in quadruplicate.*

***Supplementary Figure 5: Localization of MSC in the colon of irradiated rats treated with MSC-hy+HS-m****.*

*Representative pictures of GFP-MSC (visualized in blue) embedded in hydrogel and injected locally according to the protocol depicted in the supplementary figure 2. The GFP cells are readily detected in various quantities in the colon, but no statistical improvement of the engraftment could be highlighted when MSC-hy is combined with HS-m injection.*

*Scale bar represents 100µm.*


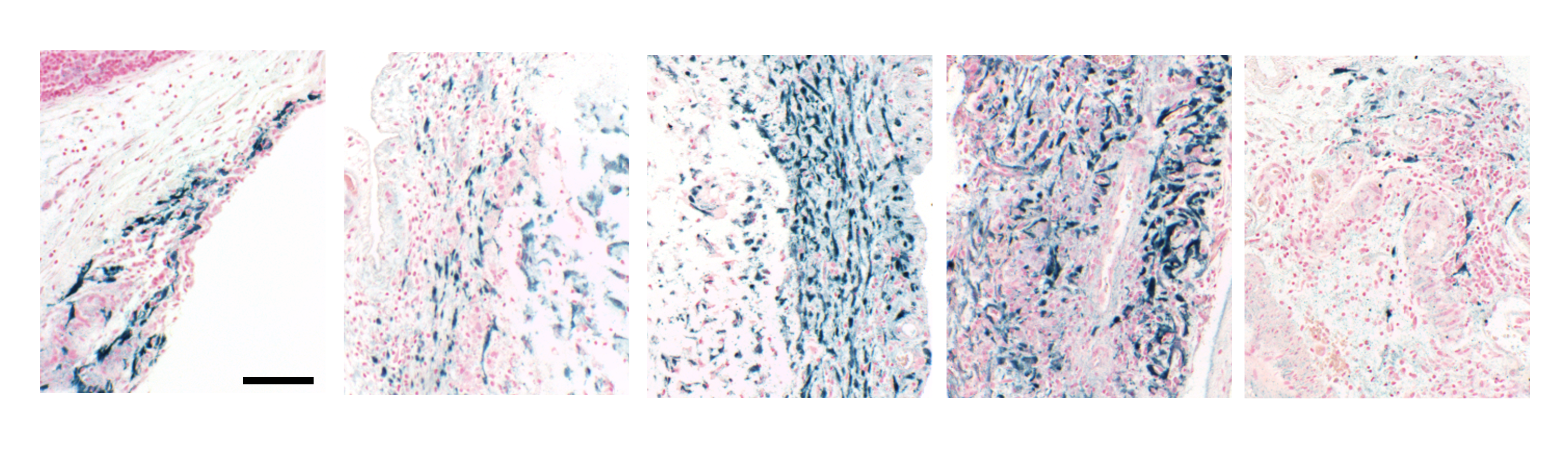


***Supplementary Figure 6: Growth factor secretion by MSC-hy with or without HS-m .***

*Proteins released from human MSC during culture within hydrogel (3D) with or without HS-m_OTR4120 polysaccharide were quantified following seven days of culture. To mimic irradiated conditions, transforming growth factor-beta (TGF-β_1_) with IL-1β were added to the culture media. On day 6, culture media was aspirated and cells washed using PBS and incubated in deprived media for 24 hours. Media was removed from cells and frozen at -80^0^C prior to analysis. Hydrogels was sonicated for DNA analysis (normalization in µg of proteins per µg of cell DNA) and media was analysed using a Luminex 27-plex system for protein analysis (Bio-rad Laboratories, UK). Experiment was realized in triplicate. ^*^p<0.05 vs MSC-Hy.*

*
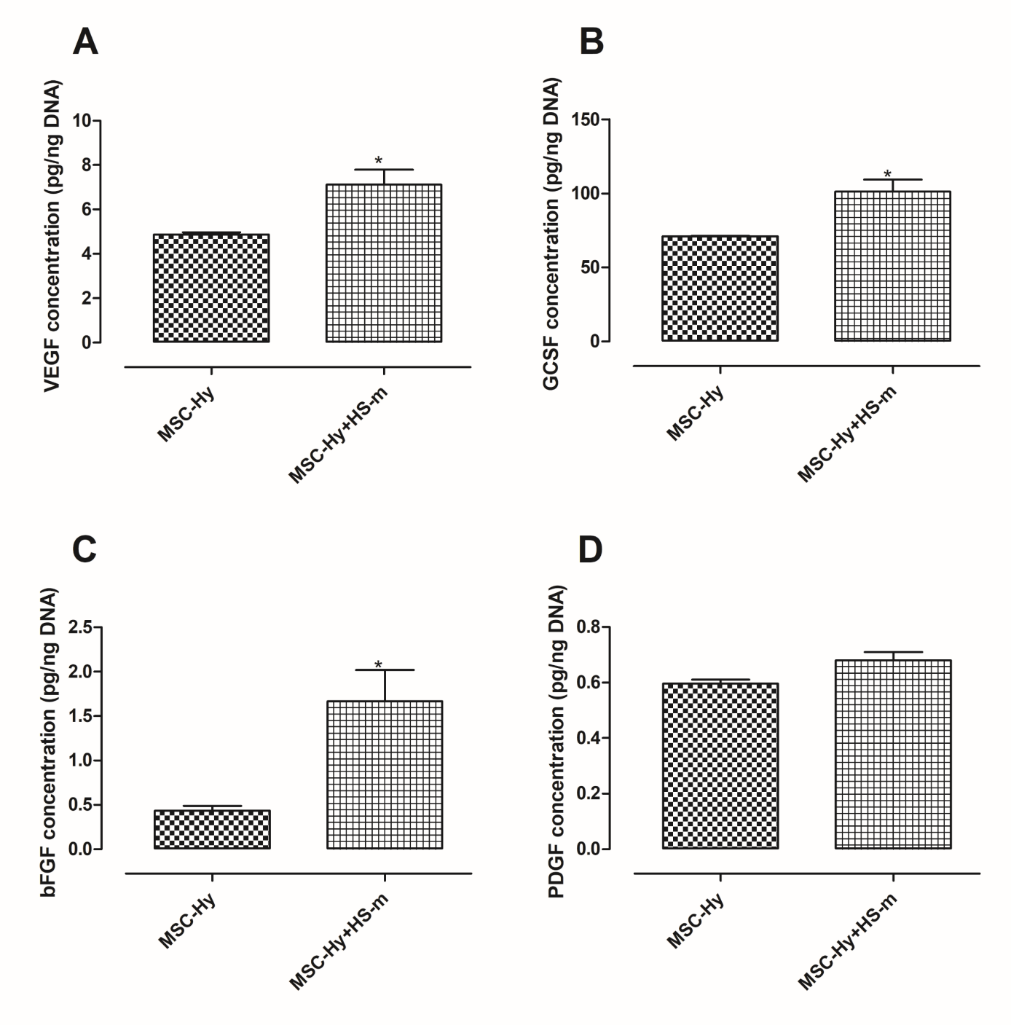
*
